# Eating behavior and psychological profile: associations between daughters with distinct eating disorders and their mothers

**DOI:** 10.1186/s12905-017-0430-y

**Published:** 2017-09-06

**Authors:** Verónica Vázquez-Velázquez, Martha Kaufer-Horwitz, Juan Pablo Méndez, Eduardo García-García, Lucy María Reidl-Martínez

**Affiliations:** 1 Obesity and Eating Disorders Clinic, Instituto Nacional de Ciencias Médicas y Nutrición Salvador Zubirán, Vasco de Quiroga 15, Colonia Belisario Domínguez Sección XVI, Tlalpan, 14080 Mexico City, Mexico; 2 Research Unit in Obesity, School of Medicine, Universidad Nacional Autónoma de México, Mexico City, Mexico; 3 School of Psychology, Universidad Nacional Autónoma de México, Mexico City, Mexico

**Keywords:** Eating disorders, Mothers, Psychological profile, Eating behavior, Association

## Abstract

**Background:**

Associations of eating behaviors and psychological profile between mothers and daughters with eating disorders exist, but it is important to dissect the influence of the mother in each specific disorder since all eating disorders must be seen or treated not as one entity. The aim of the present study was to evaluate the association of eating behavior and psychological profile between mothers and daughters with different eating disorders and a control group.

**Methods:**

The study group included young girls with anorexia nervosa (AN, *n* = 30), bulimia nervosa (BN, *n* = 30), binge eating disorder (BED, *n* = 19), and a control group of women (Non-ED, *n* = 54) together with their mothers. BMI was calculated for dyads and Eating Disorder Inventory, Beck Depression Inventory, Beck Anxiety Inventory, Toronto Alexithymia Scale and Three-Factor Eating Questionnaire were applied. The differences between dyads were tested by Student’s t test and Pearson’s correlation was used to study the association between BMI, variables of eating behavior and psychological profile in each dyad.

**Results:**

The study found significant inverse correlations between the AN dyad; some correlations between the BN dyad, and the highest positive correlations exist in BED dyad, especially in eating behavior. Finally, between the control dyads, low but significant correlations were found in the majority of cases.

**Conclusions:**

The study concluded that the associations between mothers and daughters with distinct eating disorders varied depending on the specific diagnosis of the daughter, indicating it is necessary to analyze them individually, given that there may be different implications for treatment.

## Background

Eating disorders (ED), such as anorexia nervosa (AN), bulimia nervosa (BN), and binge eating disorder (BED), are psychiatric entities that primarily affect young women and whose etiology involves multiple and distinct factors [1]. Family studies have shown that transgenerational effects exist on eating attitudes and psychopathological traits [2, 3] and that the influence of the mother is associated with the development of ED on her daughter [4]. The two main modes of influence that have been proposed to account for the association between mothers and their offspring are modelling and maternal attitudes towards their offspring’s shape, weight and eating behaviors, conveyed by verbal messages in the form of teasing, criticism, and encouragement to lose or control weight [5].

Several studies have evaluated the association between mothers and daughters with AN or BN together in the same category of ED. They conclude that an abnormal attitude toward food by the mother has been shown to be a potential risk factor for ED development in women [6] and that the mothers of women with ED (AN or BN) have more bulimic symptoms, more depression and to diet more [7, 8], but they do not present higher rates of body dissatisfaction [9].

Specifically, studies of mothers of individuals with AN have shown elevated levels of perfectionism and drive for thinness on the mother [10], and associations between them and their daughters in relation to body dissatisfaction [11]. Among patients with BN, some authors have demonstrated a strong correlation of body dissatisfaction between mothers and daughters [12], and others have found correlations between eating behavior of both [2, 7]. However, there are studies that have failed to find an association between mothers and daughters eating patterns in patients with AN or BN [12, 13]. On the other hand, it has also been shown that women with high scores in disinhibition toward food have overweight daughters [14, 15]; also that the risk for having a child with BED is higher among women with depression [16].

Although there are numerous studies in the scientific literature that indicate that associations of eating behaviors and psychological profile between mothers and daughters with eating disorders exist, they have been incorrectly systematized. This makes it difficult to dissect the characteristics of the mothers in each specific disorder since most studies treat all eating disorders as one entity, analyzing AN, BN and BED in the same group [7, 8].

To our knowledge, studies where patients with AN, BN or BED have been thoroughly and adequately diagnosed and studied separately with regard to mother’s characteristics have not been published.

Based on the above, we designed a study where associations between mothers and adolescent and young adult daughters were explored according to specific ED diagnoses; that is, AN, BN or BED as separate entities. The aim of our study was therefore to assess the association of eating behavior and the psychological profile of mothers and daughters with anorexia nervosa, bulimia nervosa, binge eating disorder, and a control group of women.

## Methods

### Design, study setting and population

A cross-sectional study was conducted in a tertiary hospital care, the National Institute of Medical Sciences and Nutrition Salvador Zubirán.

### Participants

The sampling was non probabilistic and intentional. One hundred thirty three dyads were included: 30 mother-daughter with AN dyads, 30 mother-daughter with BN dyads, 19 mother-daughter with BED and obesity dyads and 54 mother-daughter without eating disorder or obesity dyads (control group).

### Inclusion and exclusion criteria

Inclusion criteria for the diagnosis of eating disorder group were: age between 15 and 30 years, minimum level of high school education, who meet the diagnostic criteria of DSM-IV-TR for anorexia nervosa, bulimia nervosa and binge eating disorder with obesity (BMI > 30), who live with their biological mothers. For mothers, it was considered the age (between 35 and 60 years of age) and having a minimum level of high school education. For the comparison group, were the same criteria in age, education and living with their biological mother, as well as having normal weight (BMI between 18.5 - 24.9). Exclusion criteria to both mothers and daughters with ED: the presence of medical comorbidities such as diabetes mellitus type 1 and 2, thyroid problems or other pathology that compromise eating behavior and emotional status, pregnancy. For the comparison group, the daughters who had a higher rating to the cutoff of 80 on the Eating Disorder Inventory (EDI-2) [18], plus the presence of medical comorbidities: diabetes mellitus type 1 and 2, thyroid problems or other pathology that compromises eating behavior and emotional state. They were eliminated from the study anyone who does not complete the questionnaires or to decide to withdraw from the study.

### Procedure and recruitment

About 2-3 women with suspected eating disorders are seen weekly at the Eating Disorders Clinic of the Institute for investigation and initial care. Body weight and height to calculate the BMI was obtained, and it was conducted an interview with this patients to establish the diagnosis (AN, BN, BED) according to DSM-IV-TR. Women who presented an eating disorder and met the inclusion and exclusion criteria, were invited to participate in the study, along with their mothers. Written informed consent for participation in the study was obtained from each participant (mothers and daughters). A battery of psychological tests, applied separately to mothers and daughters, and the BMI was obtained for the mothers.

The group of women without eating disorders (controls), were obtained from a sample of 231 middle and high school women students (15 to 19 years old) of a private school in Mexico City and 60 women relatives and friends of students, both from a public university and a private university (except students from psychology, nutrition or medicine). According the inclusion criteria it was included dyads that agreed to participate and signed informed consent. The weight and height were obtained at the school and they answered a battery of psychological tests. The detected cases (EDI-2 > 80) were invited to attend at the Eating Disorders Clinic of the Institute to make a clinical diagnosis and, if necessary, initiate treatment.

### Ethics

The project was approved by the Ethics Committee of the National Institute of Medical Sciences and Nutrition Salvador Zubirán (Reference 1978; DIA- 201-09 / 12-1).

### Measurements

Weight and height were measured to determine the BMI scores of all women. A set of psychological tests validated for the Mexican population were applied, including: Eating Disorder Inventory (EDI-2; Garner, 1991), which involves 91 questions divided into 11 subscales: Drive for Thinness (DT), Bulimia (B), Body Dissatisfaction (BD), Ineffectiveness (I), Perfectionism (P), Interpersonal Distrust (ID), Interoceptive Awareness (IA), Maturity Fears (MF), Asceticism (A), Impulse Regulation (IR) and Social Insecurity (SI), with elevated internal consistency (Cronbach’s alpha of 0.98) [17, 18]; Beck Depression Inventory (BDI; Beck, 1988) with 21 questions, with high internal consistency (Cronbach’s alpha of .87) [19]; Beck Anxiety Inventory (BAI; Beck, 1988) with 21 questions, with high internal consistency (Cronbach’s alpha of 0.83) and high test-retest reliability (*r* = 0.75) [20]; Toronto Alexithymia Scale (TAS; Taylor, Ryan and Bagby, 1985) with 20 questions, with high internal consistency (Cronbach’s alpha of 0.87) and two factors: Difficulties in Identifying and Describing Feelings (DIDF) and Externally Oriented Thinking (EOT) [21]; and Three-Factor Eating Questionnaire (TFEQ, Stunkard & Messick, 1988) [22] with 51 questions that evaluate Dietary Restraint (DR), Disinhibition (D) and Hunger (H), with an adequate internal consistency (Cronbach’s alpha of 0.80) and a test-retest reliability by scale of: 0.84 for DR, 0.94 for D and 0.90 for H (unpublished data).

### Data analysis

Data were described using mean and standard deviation for continuous variables. The differences between dyads were tested by Student’s t test and Pearson’s correlation was used to study the association between BMI, variables of eating behavior and psychological profile in each dyad. Data were entered into Microsoft Excel and was analyzed using SPSS® data analysis and statistical software package version 19 (SPSS, Inc. USA). A *p*-value less than .05 was considered statistically significant.

## Results

The study group included 30 women with AN, 30 women with BN, 19 with BED, and 54 control women (Non-ED), together with their mothers. The average age of the patients with AN was 18.2 ± 2.9 years; patients with BN 19.1 ± 3.1; patients with BED 22.3 ± 3.1, and the control group, 19.1 ± 2.7 years. All women had completed at least a middle school education and lived with their biological mothers. Patients with AN had an average disorder duration of 2.2 ± 1.8 years; patients with BN 4.2 ± 2.9 years, and patients with BED 7.6 ± 4.5.

The average ages of the mothers were as follows: AN 45.2 ± 5.5 years, BN 47.6 ± 5.1, BED 50.3 ± 4.5, and the control group 47.4 ± 5.4 years. All mothers had completed at least middle school education.

Table 1 shows the BMI of the participants as well as scores in evaluations of eating behavior and psychological profile. As shown, the BMI of daughters with AN indicates underweight, daughters with BN and controls have normal weight, and daughters with BED have obesity class III. In the case of mothers, only the control group has normal weight, the other three groups (mothers of daughters with AN, BN and BED) are overweight It is noteworthy that no group of mothers get high scores suggesting psychopathology, in any of the scales of the instruments except the mothers of daughters with AN, which do have significant symptoms of anxiety.

**Table 1:** BMI and scores on the variables of the psychological profile and eating behavior of mothers and daughters, according to the diagnosis of daughters

	AN (n = 30)		BN (*n* = 30)		BED (*n* = 19)		Controls (*n* = 54)	
	D	M	D	M	D	M	D	M
BMI	16.9 ± 3.2	26.3 ± 4.1	22.2 ± 3.2	26.0 ± 6.5	40.5 ± 7.6	29.3 ± 4.1*	21.5 ± 1.5	23.9 ± 3.3
**Eating Disorder Inventory (EDI-2)**								
Drive for Thinness (DT) (14)	**14.3 ± 6.8**	3.4 ± 4.8	**16.7 ± +4.4**	3.2 ± 4	11.4 ± 4.2	5.4 ± 5.3	2.4 ± 3	3.1 ± 3.7
Bulimia (B) (3)	**3.2 ± 4.7**	0.2 ± .7	**9.8 ± 7**	0.1 ± .7	**5.0 ± 4.7**	1.2 ± 2.9	0.5 ± 1.3	0.2 ± .9
Body Dissatisfaction (BD) (14)	**14.4 ± 7.1**	7.0 ± 6.1	**19.1 ± 7.3**	5.7 ± 4.8	**19.6 ± 5.7**	9.7 ± 6.1	4.0 ± 4.7	5.4 ± 5.6
Ineffectiveness (I) (7)	**10.6 ± 8.6**	2.4 ± 3.7	**11.9 ± 8.5**	2.4 ± 2.3	**9.1 ± 6.2**	4.2 ± 6	0.9 ± 1.7	0.9 ± 1.5
Perfectionism (P) (11)	10.7 ± 4.2	4.5 ± 4	10.5 ± 4.1	4.4 ± 3.6	9.5 ± 3.9	6.8 ± 3.8	6.4 ± 3.8	4.7 ± 3.1
Interpersonal Distrust (ID) (7)	**8.9 ± 5.5**	3.0 ± 3.4	**7 ± 5.6**	2.7 ± 3.2	5.9 ± 5.6	5.0 ± 3.5	1.7 ± 2.1	2.1 ± 2.9
Interoceptive Awareness (IA) (11)	**13.7 ± 7.6**	3.3 ± 4.8	**13.1 ± 7.5**	2.8 ± 3.1	9.2 ± 5.7	3.8 ± 4.1	2.0 ± 3.1	1.4 ± 2.1
Maturity Fears (MF) (8)	**11.7 ± 7.1**	3.7 ± 3.2	**9.5 ± 6.9**	4.1 ± 3.1	**9.3 ± 5.9**	5.9 ± 4.4	3.9 ± 3.2	3.5 ± 3.9
Ascetism (A) (7)	**8.9 ± 5.4**	3.1 ± 2.4	**9.7 ± 5**	4.6 ± 2.9	6.8 ± 3.1	3.8 ± 2.7	2.5 ± 1.7	3.3 ± 2.3
Impulse Regulation (IR) (11)	**13.1 ± 7.4**	4.3 ± 4.7	**12.8 ± 7**	3.3 ± 3.6	7.7 ± 4.6	5.1 ± 4.4	2.1 ± 2.4	1.8 ± 2.6
Social Insecurity (SI) (6)	**10 ± 5.6**	3.1 ± 2.8	**9.4 ± 6.3**	3.0 ± 2.9	**7.3 ± 4.9**	4.1 ± 5.2	1.4 ± 1.7	1.4 ± 1.9
EDI-2 Total (EDI-2) (105)	**119.5 ± 51**	38.1 ± 26	**129.5 ± 51.7**	36.4 ± 21	**100.8 ± 31.4**	54.9 ± 31	27.7 ± 16.7	27.7 ± 18.5
**Depression (BDI) (10)**	**26.7 ± 12.1**	**14.7 ± 9**	**27.6 ± 10.8**	**11.8 ± 8**	**20.6 ± 11.7**	**16.5 ± 12.6**	5.6 ± 4.3	7.5 ± 5.5
**Anxiety (BAI) (6)**	**47.5 ± 14.8**	**16.9 ± 12.2**	**43.6 ± 14.2**	**10.9 ± 8.6**	**42.6 ± 12.3**	**15.8 ± 12.5**	**23.4 ± 10.2**	**8.0 ± 7.9**
**Toronto Alexithymia Scale (TAS)**								
Difficulties in Identifying and Describing Feelings (DIDF)	10.0 ± 4.4	27.1 ± 16	10.6 ± 5	28.0 ± 17	8.6 ± 4.4	34.2 ± 19	6.1 ± 3.2	19.7 ± 12.6
Externally Oriented Thinking (EOT)	57.6 ± 17.3	8.0 ± 4.7	54.2 ± 17.1	6.5 ± 3.5	51.2 ± 13.6	9.3 ± 5.5	29.5 ± 11.1	5.2 ± 3.7
TAS Total (TAS) (51)	26.5 ± 15.3	35.1 ± 17.3	30.0 ± 11	34.5 ± 17.9	21.4 ± 9.6	43.5 ± 20.2	9.0 ± 6.5	24.9 ± 14.8
**Three Factor Eating Questionnaire (TFEQ)**								
Dietary Restraint (DR) (14)	**16.5 ± 4.1**	7.1 ± 4.2	**15.1 ± 4.3**	8.2 ± 3.9	8.9 ± 4.8	9.8 ± 4.2	7.5 ± 4.7	10.3 ± 4.9
Disinhibition (D) (12)	4.9 ± 3.1	5.1 ± 3.4	10 ± 4	4.2 ± 3.2	11.3 ± 3.1	5.7 ± 4.1	4.9 ± 3.2	4.5 ± 3.1
Hunger (H) (11)	3.9 ± 3	3.8 ± 2.4	7.4 ± 4.4	4.2 ± 2.6	8.9 ± 3	4.2 ± 3.2	5.1 ± 2.6	3.5 ± 2.7

Data are shown as media ± standard deviation

*D* Daughters, *M* Mothers, *AN* Anorexia Nerviosa, *BN* Bulimia Nerviosa, *BED* Binge Eating Disorder

Values in **bold** suggest high and clinical scores, based on cut-off score (number in parenthesis next to each scale)

*ANOVA, *p* < 0.05, BED vs. BN and Controls

### Patients with AN and their mothers

Table 2 illustrates that significant differences existed between mothers and daughters with AN in all the variables except in Externally Oriented Thinking, Disinhibition, and Hunger. Most correlations between mother and daughter were inverse. For example, Drive to Thinness and Dietary Restraint of the daughter with Impulse Regulation of the mother; Body Dissatisfaction of the daughter with Perfectionism of the mother. Also, the lower BMI of the daughter, the higher Hunger and higher Depression of the mother. Body Dissatisfaction of the daughter was inverse to the restrictive behaviors and the psychological profile of the mother. Daughter’s Dietary Restraint inversely correlated with other variables of the mother, such as Drive to Thinness, Perfectionism, Asceticism and total EDI-2 score.

**Table 2:** Comparison and correlations of BMI, eating behavior and psychological profile in patients with anorexia nervosa and their mothers

	AN		r	Statistically significant correlations with other scales of mother ^§^
	Daughters	Mothers		
BMI	16.9 ± 3.2	26.3 ± 4.1**	.29	H (−.37), BDI (−.43)
Eating Disorder Inventory (EDI-2)				
Drive for Thinness (DT)	14.3 ± 6.8	3.4 ± 4.8**	−.37*	P (−.39),MF (−.44), A (−.42), IR (−.60), EDI-2 (−.43)
Bulimia (B)	3.2 ± 4.7	0.2 ± 0.7*	−.18	
Body Dissatisfaction (BD)	14.4 ± 7.1	7 ± 6.1**	−.13	P (−.62), MF (−.36), EDI-2 (−.43), DR (−.37)
Ineffectiveness (I)	10.6 ± 8.6	2.4 ± 3.7**	.09	MF (−.36)
Perfectionism (P)	10.7 ± 4.2	4.5 ± 4**	−.41*	BD (−.39)
Interpersonal Distrust (ID)	8.9 ± 5.5	3.0 ± 3.5**	.19	
Interoceptive Awareness (IA)	13.7 ± 7.6	3.3 ± 4.8**	.13	P (−.39)
Maturity Fears (MF)	11.7 ± 7.1	3.7 ± 3.2**	−.32	P (−.38), EOT (−.45)
Ascetism (A)	8.9 ± 5.4	3.1 ± 2.4**	−.11	P (−.49)
Impulse Regulation (IR)	13.1 ± 7.4	4.3 ± 4.7**	−.12	P (−.54)
Social Insecurity (SI)	10 ± 5.6	3.1 ± 2.8**	−.12	MF (−.36)
EDI-2 Total (EDI-2)	119.5 ± 51	38.1 ± 26**	−.28	P (−.52), MF (−.39)
Depression (BDI)	26.7 ± 12.1	14.7 ± 9**	−.07	P (−.42)
Anxiety (BAI)	26.5 ± 15.3	16.9 ± 12.2*	.30	
Toronto Alexithymia Scale (TAS)				
Difficulties in Identifying and Describing Feelings (DIDF)	47.5 ± 14.8	27.1 ± 16**	.12	MF (−.50)
Externally Oriented Thinking (EOT)	10.0 ± 4.4	8.0 ± 4.7	.05	B (.48)
TAS Total (TAS)	57.6 ± 17.3	35.1 ± 17.3**	.06	P (−.36), MF (−.45)
Three Factor Eating Questionnaire (TFEQ)				
Dietary Restraint (DR)	16.5 ± 4.1	7.1 ± 4.2**	−.20	DT (−.42), P (−.50), A (−.55), IR (−.63), EDI-2 (−.45)
Disinhibition (D)	4.9 ± 3.1	5.1 ± 3.4	−.16	DT (−.39)
Hunger (H)	3.9 ± 3.0	3.8 ± 2.4	.29	I (.37)

Data are shown as media ± standard deviation

* *p* < .05, ** *p* < .001

^**§**^ Correlations between the daughter’s score on this scale and the mother’s score on other scales of the questionnaires

### Patients with BN and their mothers

In the patients with BN and their mothers, statistically significant differences were found in all variables (Table 3), including BMI. It was found that Difficulties in Identifying and Describing Feelings and Alexithymia in the daughter inversely correlated to Externally Oriented Thinking of the mother. Also, we found that daughter’s difficulty for Impulse Regulation positively correlates with mother’s Anxiety, and that daughter’s Disinhibition inversely correlated with mother’s Dietary Restraint.

**Table 3:** Comparison and correlations of BMI, eating behavior and psychological profile in patients with bulimia nervosa and their mothers

	BN		r	Statistically significant correlations with other scales of mother^§^
	Daughters	Mothers		
BMI	22.2 ± 3.2	26.0 ± 6.5**	.51**	BD (.39)
Eating Disorder Inventory (EDI-2)				
Drive for Thinness (DT)	16.7 ± +4.4	3.4 ± 4.1**	.18	
Bulimia (B)	9.8 ± 7.0	0.3 ± 1.0**	.07	
Body Dissatisfaction (BD)	19.1 ± 7.3	6.3 ± 5.9**	−.01	
Ineffectiveness (I)	11.9 ± 8.5	2.3 ± 2.3**	−.22	
Perfectionism (P)	10.5 ± 4.1	4.4 ± 3.6**	.05	
Interpersonal Distrust (ID)	7 ± 5.6	2.7 ± 3.2**	.20	
Interoceptive Awareness (IA)	13.1 ± 7.5	2.9 ± 3.1**	.04	
Maturity Fears (MF)	9.5 ± 6.9	4.2 ± 3.2**	.15	
Ascetism (A)	9.7 ± 5	4.6 ± 2.8**	.03	SI (.39)
Impulse Regulation (IR)	12.8 ± 7	3.3 ± 3.5**	.19	BAI (.39)
Social Insecurity (SI)	9.4 ± 6.3	3 ± 2.9**	.06	
EDI-2 Total (EDI-2)	129.5 ± 51.7	37.5 ± 21.7**	.10	
Depression (BDI)	27.6 ± 10.8	12.0 ± 8**	.27	
Anxiety (BAI)	30.0 ± 11	11.6 ± 9.3**	.12	
Toronto Alexithymia Scale (TAS)				
Difficulties in Identifying and Describing Feelings (DIDF)	43.6 ± 14.2	27.8 ± 16.7**	.19	EOT (−.53)
Externally Oriented Thinking (EOT)	10.6 ± 5	6.4 ± 3.5*	−.16	
TAS Total (TAS)	54.2 ± 17.1	34.3 ± 17.7**	.03	EOT (−.49)
Three Factor Eating Questionnaire (TFEQ)				
Dietary Restraint (DR)	15.1 ± 4.3	8.3 ± 3.9**	.21	
Disinhibition (D)	10 ± 4.0	4.5 ± 3.5**	−.02	DR (−.38)
Hunger (H)	7.4 ± 4.4	4.3 ± 2.6*	−.14	

Data are shown as media ± standard deviation

* p < .05, ** p < .001

^**§**^ Correlations between the daughter’s score on this scale and the mother’s score on other scales of the questionnaires

### Patients with BED and their mothers

Significant differences were found between patients with BED and their mothers in BMI and in all scales except Interpersonal Distrust, Depression, Anxiety, the scales and total of TAS and Dietary Restraint (Table 4). The only inverse correlation was between daughter’s BMI and mother’s Drive for Thinness.

**Table 4:** Comparison and correlations of BMI, eating behavior and psychological profile in patients with binge eating disorder and their mothers

	BED		r	Statistically significant correlations with other scales of mother^§^
	Daughters	Mothers		
BMI	40.5 ± 7.6	29.3 ± 4.1**	.20	DT (−.46)
Eating Disorder Inventory (EDI-2)				
Drive for Thinness (DT)	11.4 ± 4.2	5.4 ± 5.3**	.37	
Bulimia (B)	5.0 ± 4.7	1.2 ± 3.0*	−.25	
Body Dissatisfaction (BD)	19.6 ± 5.7	9.7 ± 6.1**	.24	
Ineffectiveness (I)	9.1 ± 6.2	4.2 ± 6*	.41	
Perfectionism (P)	9.5 ± 3.9	6.8 ± 3.8*	.22	
Interpersonal Distrust (ID)	5.9 ± 5.6	5.0 ± 3.5	.42	
Interoceptive Awareness (IA)	9.2 ± 5.7	3.8 ± 4.1**	.30	
Maturity Fears (MF)	9.3 ± 5.9	5.9 ± 4.4*	.55*	I (.55), IA (.47)
Ascetism (A)	6.8 ± 3.1	3.8 ± 2.7*	.25	P (.48)
Impulse Regulation (IR)	7.7 ± 4.6	5.1 ± 4.4*	.45	
Social Insecurity (SI)	7.3 ± 4.9	4.1 ± 5.2*	.38	
EDI-2 Total (EDI-2)	100.8 ± 31.4	54.9 ± 31**	.28	IA (.47)
Depression (BDI)	20.6 ± 11.7	16.5 ± 12.6	.34	B (.50), I (.46), IA (.61), EDI-2 (.49)
Anxiety (BAI)	21.4 ± 9.6	15.8 ± 12.5	.32	
Toronto Alexithymia Scale (TAS)				
Difficulties in Identifying and Describing Feelings (DIDF)	42.6 ± 12.3	34.2 ± 19.1	.16	ID (.50), EOT (.58)
Externally Oriented Thinking (EOT)	8.6 ± 4.4	9.3 ± 5.5	.02	
TAS Total (TAS)	51.2 ± 13.6	43.5 ± 20.2	.25	EOT (.53)
Three Factor Eating Questionnaire (TFEQ)				
Dietary Restraint (DR)	8.9 ± 4.8	9.8 ± 4.2	.49*	ID (−.49)
Disinhibition (D)	11.3 ± 3.1	5.7 ± 4.1**	.45	H (.50)
Hunger (H)	8.9 ± 3.0	4.2 ± 3.2**	.15	

Data are shown as media ± standard deviation

* p < .05, ** p < .001

^**§**^ Correlations between the daughter’s score on this scale and the mother’s score on other scales of the questionnaires

There were positive correlations between mother and daughter in Dietary Restraint. Daughter’s depression was associated with Bulimia, Ineffectiveness, Interoceptive Awareness and EDI-2 of the mother. The BED daughter’s abnormal eating behavior of Disinhibition significantly correlated with mother’s Hunger. Also, Alexithymia in the daughter correlated to Externally Oriented Thinking of the mother.

### Control group and their mothers

Table 5 shows that, as we expected, there were more similarities between the controls and their mothers than in the other three groups. Differences were only found in BMI (although the correlation was significant), Perfectionism, Asceticism, Depression, Dietary Restraint and Hunger. Significant correlations between mothers and daughters were found in Bulimia, Ineffectiveness and Disinhibition.

**Table 5:** Comparison and correlations of BMI, eating behavior and psychological profile between daughters and their mothers in the Non-ED group

	Non-ED		r	Statistically significant correlations with other scales of mother^§^
	Daughters	Mothers		
BMI	21.5 ± 1.5	23.9 ± 3.3**	.33*	
Eating Disorder Inventory (EDI-2)				
Drive for Thinness (DT)	2.4 ± 3.0	3.1 ± 3.7	.02	
Bulimia (B)	0.5 ± 1.3	0.2 ± 0.9	.47**	
Body Dissatisfaction (BD)	4.0 ± 4.7	5.4 ± 5.6	.24	
Ineffectiveness (I)	0.9 ± 1.7	0.9 ± 1.5	.45**	B (.31), P (.34), IA (.34), EDI-2 (.36)
Perfectionism (P)	6.4 ± 3.8	4.7 ± 3.1*	.13	
Interpersonal Distrust (ID)	1.7 ± 2.1	2.1 ± 2.9	−.20	P (.30)
Interoceptive Awareness (IA)	2.0 ± 3.1	1.4 ± 2.1	−.03	
Maturity Fears (MF)	3.9 ± 3.2	3.5 ± 4	.18	B (.32)
Ascetism (A)	2.5 ± 1.7	3.3 ± 2.3*	.21	TAS (−.27)
Impulse Regulation (IR)	2.1 ± 2.4	1.8 ± 2.6	.17	I (.30), SI (.27)
Social Insecurity (SI)	1.4 ± 1.7	1.4 ± 2	.05	P (.34)
EDI-2 Total (EDI-2)	27.7 ± 16.7	27.7 ± 18.5	.12	
Depression (BDI)	5.6 ± 4.3	7.5 ± 5.5*	.11	IR (.34)
Anxiety (BAI)	9.0 ± 6.5	8.0 ± 7.9	.19	
Toronto Alexithymia Scale (TAS)				
Difficulties in Identifying and Describing Feelings (DIDF)	23.4 ± 10.2	19.7 ± 12.6	.12	P (.28), A (.31)
Externally Oriented Thinking (EOT)	6.1 ± 3.2	5.2 ± 3.7	.03	H (.30)
TAS Total (TAS)	29.5 ± 11.1	24.9 ± 14.8	.07	P (.32), A (.33)
Three Factor Eating Questionnaire (TFEQ)				
Dietary Restraint (DR)	7.5 ± 4.7	10.3 ± 4.9**	.24	
Disinhibition (D)	4.9 ± 3.2	4.5 ± 3.1	.32*	H (.39), B (.30)
Hunger (H)	5.1 ± 2.6	3.5 ± 2.7**	.20	DT (−.27), BD (−.31)

Data are shown as media ± standard deviation

* p < .05, ** p < .001

^**§**^Correlations between the daughter’s score on this scale and the mother’s score on other scales of the questionnaires

## Discussion

We expected to find associations between mother and daughter; however we wanted to know how the associations in these pathological entities were. Associations between mothers and daughters with distinct eating disorders varied depending on the specific diagnosis of the daughter.

The inverse correlations between mother and daughter with AN may suggest the fact that in anorexia nervosa, traditionally reported in the past, that the conflict lies in the poor identification with the mother or in the rebellion of the daughter against being like her mother. While processes of imitation, internalization and identification are completely consistent with normal development, in anorexia nervosa the eating disorder is probably the only means that allows the daughter to initiate this individualization process which has been barred for her [23–25]. The fact that the lower the BMI of the daughter, the higher the symptoms of depression in the mother, may reflect the relation between the gravity of the illness of the daughter and the depression of the mother, as some authors have demonstrated [26], or that the depression of the mother predates the appearance of the AN and influences said AN development, as other authors have demonstrated [27]. Even though, the origin of this relation cannot be demostrated by the present study. On the other hand, our study did not find association of depression of mothers with asceticism of daughters, as has been found by Bachar et al. [28]. We do not know if the duration of AN has to do with this lack of association (since the duration of the disease in our patients was 2 years). Furthermore, as has been reported by Benninghoven et al. [12], we found no positive association between body dissatisfaction between mother and daughter. Even when this condition, together with drive for thinness, was present in the daughter, the symptoms of restriction, psychopathology, perfectionism, and asceticism in the mother were lower than in daughter. McKinley [29], like us, found a negative association between mother’s body shame and daughter’s body esteem. But in our study, we only found that mother’s body dissatisfaction was inversely associated with daughter’s perfectionism. To our knowledge, the finding of inverse correlations in this group had not been reported by other authors, and highlights the fact that in AN, the behaviors of the daughter are contrary to those of the mother.

Regarding the association between mothers and daughters with BN, the most relevant correlations showed that the higher the disinhibition of the daughter, the lower the dietary restraint of the mother, which means that both the daughters and the mothers presented difficulty restricting their eating (inverse correlation between disinhibition and dietary restraint). Stunkard and Messick [22] had already determined that disinhibition of the mothers regarding food is related with free access to food and disinhibition in the daughters, which is a characteristic behavior of BN. However, our study did not find this direct association in disinhibition. On the other hand, contrary to that found by Benninghoven et al. [12], we found no association between body dissatisfaction between mother and daughter. The few correlations between this dyad may require a deeper analysis, being necessary to know the role of the father or siblings in the development of BN, as founded by some authors that paternal teasing is a significant predictor of bulimic behaviors [30–32]. This issue requires more research.

In BED dyad, we found that hunger of the mother was significantly associated with disinhibition in the daughter; and that greater BMI of the daughter was associated with lower drive to thinness of the mother. To our knowledge this has not been reported by other authors in patients. The relevant aspect of the associations in this dyad is that the abnormal eating behavior of the daughter is associated in turn with an abnormal eating behavior as well as depression, anxiety and alexithymia in the mothers. Several authors have demonstrated, in other populations without diagnosis of ED, that mothers preoccupied with weight and food have daughters who are dissatisfied with their bodies, and who go through alternating periods of restriction and binging [22, 33–37].

Despite the fact that it was not the purpose of this study, mothers of this group had higher BMI than the rest of the groups: It is possible that some of their responses reflected their desire to have a healthier weight. On the other hand, Hudson et al. [38] demonstrated a higher probability that family member of patients with BED would have a higher risk of presenting obesity, especially morbid obesity, and in turn BED. To our knowledge, this finding has not been previously reported, given the lack of modelling studies between mothers and daughters with BED.

Regarding correlations between mothers and daughters without ED, they indicate that associations evidently exist between the two, given the nature of the mother-daughter relationship. These results are compatible with those found by Boschi et al. [39] in the control group of their study, where they found significant correlations between mothers and daughters in scales of bulimia, disinhibition and the use of diets. It was interesting to corroborate that mothers of our control group did not have a weight problem (normal BMI, not overweight or obese) in comparison with the other groups. Despite the design of the present study, it is possible that the mothers of this group have a positive influence on their daughters, who were deliberately chosen in this study within normal weight ranges and without any eating pathologies, as demonstrated by Hahn-Smith and Smith [40].

Given the cross-sectional nature of our study, a causal relationship cannot be established. However, it appears to be a first step toward establishing a possible modeling effect, given the lack of information according to specific ED. Likewise, our results do not establish whether mother’s psychological profile precedes the appearance of one of the specific ED in their daughters; however, there are some traits of the psychological profile that are relatively stable across time (depressive or compulsive personality traits) that may be present before the onset of the ED.

To our knowledge, there is no study in the literature that has described, compared and correlated eating behavior and psychological profile between mothers and adolescent or young daughters separately according to specific ED diagnoses, especially in the group of patients with BED, of which there is little literature on this subject.

The clinical implications of this study are the need to recognize that the mother, who usually accompanies daughters in the pathology and is the primary caregiver, may also present some problems similar to those of her daughters in relation to food, body image and emotional state (prior to the onset of the ED or in consequence). Such problems could interfere in their relationship with their daughters, but also in their quality of life. As in other pathologies (such as schizophrenia, cancer) [41, 42], attention to the patient’s caregiver has been relevant, since it can have an impact (positive or negative) in the treatment of patients; and in ED could represent an area of opportunity to improve directly or indirectly the physical and mental health of the daughter.

## Conclusions

Associations between mothers and daughters with distinct eating disorders varied depending the diagnosis of the daughter.

## Abbreviations

A: Asceticism
AN: Anorexia nervosa
B: Bulimia
BAI: Beck anxiety inventory
BD: Body dissatisfaction
BDI: Beck depression inventory
BED: Binge eating disorder
BMI: Body mass index
BN: Bulimia nervosa
D: Disinhibition
DIDF: Difficulties in identifying and describing feelings
DR: Dietary restraint
DSM-IV-TR: Diagnostic and statistical manual of mental disorders, 4th. Ed., text revision
DT: Drive for thinness
ED: Eating disorders
EDI-2: Eating disorder inventory 2
EOT: Externally oriented thinking (EOT)
H: Hunger
I: Ineffectiveness
IA: Interoceptive awareness
ID: Interpersonal distrust
IR: Impulse regulation
MF: Maturity fears
Non-ED: Non eating disorder
P: Perfectionism
SI: Social insecurity
TAS: Toronto alexithymia scale
TFEQ: Three-factor eating questionnaire

## References

[1] American Psychiatric Association (2002). Manual diagnóstico y estadístico de los trastornos mentales (DSM-IV-TR 4ta. ed. Texto Revisado).

[2] Steiger H, Stotland S, Trottier J, Ghadirian AM (1996). Familial eating concerns and psychopathological traits: causal implications of transgenerational effects. Int J Eat Disord.

[3] Bulik CM, Reba L, Siega-Riz A, Reichborn-Kiennerud T (2005). Anorexia nervosa: definition, epidemiology, and cycle of risk. Int J Eat Disord.

[4] McCabe MP, Ricciardelli LA (2005). A prospective study of pressures from parents, peers, and the media on extreme weight change behaviors among adolescent boys and girls. Behav Res Ther.

[5] Rodgers R, Chabrol H (2009). Parental attitudes, body image disturbance and disordered eating amongst adolescents and young adults: a review. Eur Eat Disord Rev.

[6] Yanez AM, Peix MA, Atserias N, Arnau A, Brug J (2007). Association of eating attitudes between teenage girls and their parents. Int J Soc Psychiatry.

[7] García de Amusquibar AM, De Simone DJ (2003). Some features of mothers of patients with eating disorders. Eat Weight Disord.

[8] Pike KM, Rodin J (1991). Mothers, daughters, and disordered eating. J Abnorm Psychol.

[9] Cobelo AW, de Chermont Prochnik Estima C, Nakano EY, Conti MA, Cordás TA (2010). Body image dissatisfaction and eating symptoms in mothers of adolescents with eating disorders. Eat Weight Disord.

[10] Woodside DB, Bulik CM, Halmi KA, Fichter MM, Kaplan A, Berrettini WH (2002). Personality, perfectionism, and attitudes toward eating in parents of individuals with eating disorders. Int J Eat Disord.

[11] Bönsch C, Raml E, Seiwald M, Rathner G (1993). Body image of anorexic girls, their mothers and sisters: a controlled study. Psychother Psychosom Med Psychol.

[12] Benninghoven D, Tetsch N, Kunzendorf S, Jantschek G (2007). Body image in patients with eating disorders and their mothers, and the role of family functioning. Compr Psychiatry.

[13] Kanakis DM, Thelen MH (1995). Parental variables associated with bulimia nervosa. Addict Behav.

[14] Cutting TM, Fisher JO, Grimm-Thomas K, Birch LL (1999). Like mother, like daughter: familial patterns of overweight are mediated by mothers’ dietary disinhibition. Am J Clin Nutr.

[15] Johannsen DL, Johannsen NM, Specker BL (2000). Influence of parents’ eating behaviors and child feeding practices on children’s weight status. Obesity.

[16] Fairburn CG, Doll HA, Welch SL, Hay PJ, Davies BA, O’Connor ME (1998). Risk factors for binge eating disorder. A community-based, case-control study. Arch Gen Psychiatry.

[17] Garner DM (1991). Eating disorder Inventory-2. Professional manual.

[18] García-García E, Vázquez-Velázquez V, López-Alvarenga JC, Arcila-Martínez D (2003). Validez interna y utilidad diagnóstica del Eating Disorder Inventory en mujeres mexicanas. Salud Pública Mex.

[19] Jurado S, Villegas ME, Méndez L, Rodríguez F, Loperena V, Varela R (1998). La estandarización del Inventario de Depresión de Beck para los residentes de la ciudad de México. Salud Ment.

[20] Robles R, Varela R, Jurado S, Páez F (2001). Versión mexicana del Inventario de Ansiedad de Beck: Propiedades psicométricas. Revista Mexicana Psicología.

[21] Pérez-Rincón H, Cortés J, Ortíz S, Peña J, Ruíz J, Díaz-Martínez A (1997). Validación y estandarización de la versión española de la Escala Modificada de Alexitimia de Toronto. Salud Ment.

[22] Stunkard AJ, Messick S (1998). Eating inventory manual.

[23] Bruch H (1973). Eating disorders: obesity, anorexia nervosa and the person within.

[24] Mahler M (1987). El desarrollo psicoafectivo e intelectual del niño.

[25] Sherkow SP, Kamens SR, Megyes M, Loewenthal L (2009). A clinical study of the intergenerational transmission of eating disorders from mothers to daughters. Psychoanal Study Child.

[26] Ravi S, Forsberg S, Fitzpatrick K, Lock J (2009). Is there a relationship between parental self-reported psychopathology and symptom severity in adolescents with anorexia nervosa?. Eat Disord.

[27] Minuchin S, Baker L, Rosman BL, Liebman R, Milman L, Todd TC (1975). A conceptual model of psychosomatic illness in children. Family organization and family therapy. Arch Gen Psychiatry.

[28] Bachar E, Kanyas K, Latzer Y, Canetti L, Bonne O, Lerer B (2008). Depressive tendencies and lower levels of self-sacrifice in mothers, and selflessness in their anorexic daughters. Eur Eat Disord Rev.

[29] McKinley NM (1999). Women and objectified body consciousness: mothers’ and daughters’ body experience in cultural, developmental, and familial context. Dev Psychol.

[30] Haslam M, Mountford V, Meyer C, Waller G (2008). Invalidating childhood environments in anorexia and bulimia nervosa. Eating Behav.

[31] Keery H, Boutelle KN, Van den Berg P, Thompson K (2005). The impact of appearance-related teasing by family members. J Adolesc Health.

[32] Field AE, Javaras KM, Aneja P, Kitos N, Camargo CA, Taylor B (2008). Family, peer, and media predictors of becoming eating disordered. Arch Pediatr Adolesc Med.

[33] Anschutz DJ, Kanters LJ, Van Strien T, Vermulst AA, Engels RC (2008). Maternal behaviors and restrained eating and body dissatisfaction in young children. Int J Eat Disord.

[34] Baker CW, Whisman MA, Brownell KD (2000). Studying intergenerational transmission of eating attitudes and behaviors: methodological and conceptual questions. Health Psychol.

[35] Cooley E, Toray T, Wang MC, Valdez NN (2008). Maternal effects on daughters’ eating pathology and body image. Eating Behav.

[36] Hirokane K, Tokumura M, Nanri S, Kimura K, Saito I (2005). Influences of mothers’ dieting behaviors on their junior high school daughters. Eat Weight Disord.

[37] Neumark-Sztainer D, Bauer KW, Friend S, Hannan PJ, Story M, Berge JM (2010). Family weight talk and dieting: how much do they matter for body dissatisfaction and disordered eating behaviors in adolescent girls?. J Adolesc Health.

[38] Hudson JI, Lalonde JK, Berry JM, Pindyck LJ, Bulik CM, Crow SJ (2006). Binge-eating disorder as a distinct familial phenotype in obese individuals. Arch Gen Psychiatry.

[39] Boschi V, Muscariello E, Maresca I, Ricciardi Lo Schiavo F, Tranchese V (2010). Assessment of eating behaviour in young women requesting nutritional counselling and their mothers. Eat Weight Disord.

[40] Hahn-Smith AM, Smith JE (2001). The positive influence of maternal identification on body image, eating attitudes, and self-esteem of Hispanic and Anglo girls. Int J Eat Disord.

[41] Awad AG, Voruganti LN (2008). The burden of schizophrenia on caregivers: a review. PharmacoEconomics.

[42] Louis JM, Adams L, Brown TL (2017). Treating depression in the caregivers of cancer patients. J Psychol Clin Psychiatry.

